# Clinical Utility and Outcomes of Repeat Computed Tomography Pulmonary Angiography in Suspected Pulmonary Embolism: A Retrospective Cohort Study

**DOI:** 10.7759/cureus.85845

**Published:** 2025-06-12

**Authors:** Bayan A Albeladi, Sarah M Althagafi, Atheer K Alharbi, Ghaday H Kasem, Husain Y Ebrahim

**Affiliations:** 1 Radiology, King Salman Medical City, Medina, SAU; 2 Radiology, King Fahd Hospital, Medina, SAU; 3 General Practice, Dammam Medical Complex, Dammam, SAU

**Keywords:** computed tomography pulmonary angiography, contrast-related adverse events, d-dimer, diagnostic accuracy, imaging utilization, radiological risks, repeat ctpa, risk stratification, venous thromboembolism

## Abstract

Background: Pulmonary embolism (PE) remains a significant cause of morbidity and mortality. While computed tomography pulmonary angiography (CTPA) is the diagnostic gold standard, uncertainty persists in some patients after a negative or inconclusive scan. The role of repeat CTPA and the utility of D-dimer testing in this context are not well defined.

Objectives: The objectives of this study are to evaluate the prevalence and severity of PE detected on repeat CTPA, assess the diagnostic performance of D-dimer testing in repeat evaluations, and examine the clinical outcomes and resource implications associated with repeat imaging.

Methods: This retrospective cohort study was conducted across public tertiary care hospitals in Eastern Province, Saudi Arabia, from January 2022 to December 2023. Adult patients (≥18 years) presenting to the emergency department with suspected PE who underwent initial CTPA and had a repeat visit within 30 days were included. Patients undergoing repeat CTPA comprised the primary analysis group. Data included demographics, clinical risk factors, D-dimer levels, CTPA findings, and outcomes. Diagnostic accuracy metrics were calculated for D-dimer. Statistical analyses compared patients with and without repeat imaging.

Results: Out of 412 patients evaluated for suspected PE, 61 (14.8%) underwent repeat CTPA. These patients were older and more likely to have cancer, a history of venous thromboembolism (VTE), and elevated Wells scores. PE was detected in 34.4% (n=21) of the repeat scans compared to 14.3% (n=59) of the initial scans (P < 0.001). Repeat imaging showed more frequent right ventricular strain (14.8%; n=9) and incidental findings (18.0%; n=11). D-dimer testing remained highly sensitive (>95%) but had low specificity; however, its positive predictive value increased during repeat visits (38.2% (21 of 55) vs. 19.3% (57 of 295)). Repeat CTPA was associated with higher hospital admission rates (55.7% (n=34) vs. 16.8% (n=59), P < 0.001) and ICU admission rates (9.8% (n=6) vs. 1.4% (n=5), P = 0.002), more frequent contrast-related adverse events (6.6% (n=4 ) vs. 1.1% (n=4), P = 0.017), and significant changes in clinical management (42.6%; n=26).

Conclusions: Repeat CTPA identifies a meaningful proportion of missed or newly developed PEs, particularly in high-risk patients. However, its benefits must be weighed against increased adverse events and healthcare resource use. Improved risk stratification and more targeted use of D-dimer testing could help optimize repeat imaging decisions and reduce unnecessary scans.

## Introduction

Pulmonary embolism (PE) represents a significant cause of morbidity and mortality worldwide. Prompt and accurate diagnosis is critical, as timely initiation of anticoagulation therapy reduces the risk of adverse outcomes, including recurrent thromboembolism and death [1,2]. Computed tomography pulmonary angiography (CTPA) has emerged as the diagnostic gold standard in emergency settings, owing to its high sensitivity, specificity, and widespread availability [1].

Despite advances in diagnostic imaging, clinical uncertainty often persists after an initial negative or inconclusive CTPA, particularly in patients presenting with recurrent or ongoing symptoms. In such cases, repeat CTPA is frequently performed, yet its clinical utility remains inadequately characterized [3,4]. Repeat imaging carries inherent risks, including radiation exposure, contrast-induced nephropathy, allergic reactions, and increased healthcare costs [1-4]. The diagnostic yield of repeat CTPA, however, varies widely across studies, and factors influencing the decision to repeat imaging, such as symptom progression, risk factors, and biomarker levels, are not well defined.

D-dimer testing, a fibrin degradation product assay, is routinely used in conjunction with clinical prediction rules to stratify patients’ risk of PE and guide imaging decisions. Although highly sensitive, its specificity is limited, particularly in populations with recent illness, inflammation, or prior thromboembolism, leading to frequent false-positive results and potentially unnecessary imaging [5,6]. This diagnostic dilemma is amplified in patients undergoing repeat evaluations, where the predictive value of D-dimer remains unclear.

Given these considerations, there is a pressing need to better understand the role and outcomes of repeat pulmonary angiography and D-dimer testing in the emergency evaluation of suspected PE. This study aims to characterize the prevalence and severity of PE detected on repeat CTPA, assess the diagnostic performance of D-dimer testing in this context, and evaluate the impact of repeat imaging on patient outcomes and resource utilization.

## Materials and methods

Study design and setting

This was a retrospective cohort study based on data from the unified electronic system of the Ministry of Health (MOH), which aggregates patient information across all public tertiary care hospitals in the Eastern Province of Saudi Arabia, between January 1, 2022, and December 31, 2023. The study included adult patients (≥18 years) who presented to the emergency department (ED) with suspected PE and underwent CTPA as part of their diagnostic workup. The study was approved by the Standing Committee for Sabbatical Leaves, Publication and Research Ethics, Ministry of Health, Saudi Arabia (approval number: REC-2021-17793), with a waiver of informed consent due to the retrospective nature of the analysis.

Patient selection

Patients were identified using electronic medical records based on clinical documentation and radiology order codes for CTPA performed in the ED. Patients were included if they underwent an initial CTPA for suspected PE and had a subsequent ED revisit within 30 days. Among these, patients who underwent repeat CTPA during the second visit formed the primary analysis group. Patients with incomplete records, missing imaging studies, or known chronic PE were excluded.

Data collection

Clinical data were extracted by trained reviewers using a standardized abstraction form. Collected variables included age, sex, BMI, smoking status, comorbidities (including hypertension, diabetes mellitus, active cancer, and history of venous thromboembolism), initial and repeat visit symptoms, Wells score, D-dimer results, and details of CTPA findings. The presence of PE was categorized anatomically (main, segmental, or subsegmental arteries). Additional findings (e.g., pneumonia, effusion, or atelectasis) and signs of right ventricular (RV) strain were recorded.

The time between visits, anticoagulation status, clinical outcomes (admission, ICU transfer, mortality), contrast-related adverse events, and healthcare utilization (including cost estimates) were also collected.

Diagnostic and imaging definitions

A positive D-dimer was defined as a value above the institution-specific upper limit of normal (>500 ng/mL fibrinogen equivalent units). Right ventricular (RV) strain on CTPA was defined by standard radiologic criteria (e.g., RV-to-left ventricular (LV) diameter ratio >1.0). All imaging reports were reviewed by board-certified radiologists, with independent revalidation of a random sample of 10% for interobserver agreement.

Outcomes

The primary outcome was the presence of PE on repeat CTPA. Secondary outcomes included changes in clinical management resulting from repeat imaging, the diagnostic performance of D-dimer testing, hospital or ICU admission, 30-day mortality, and imaging-related adverse events. Healthcare cost was estimated based on billing data, including imaging, laboratory, and inpatient services.

Statistical analysis

Continuous variables were reported as means with standard deviations (SD) or medians with interquartile ranges (IQR), depending on distribution. Categorical variables were reported as frequencies and percentages. Between-group comparisons were performed using the Student’s t-test or the Mann-Whitney U test for continuous variables and the chi-square or Fisher’s exact test for categorical variables.

Diagnostic performance of D-dimer (sensitivity, specificity, negative predictive value (NPV), and positive predictive value (PPV)) was calculated using standard 2×2 tables. A P value <0.05 was considered statistically significant. All analyses were performed using IBM SPSS Statistics for Windows, version 26.0 (Released 2018; IBM Corp., Armonk, New York, United States).

## Results

Patient characteristics

A total of 412 patients were evaluated for suspected PE in the ED during the study period. Of these, 61 patients (14.8%) underwent repeat CTPA during a subsequent ED visit within 30 days, while 351 patients (85.2%) did not (Table 1).

**Table 1:** Baseline characteristics of the study population (N=412) CTPA: computed tomography pulmonary angiography; BMI: body mass index; VTE: venous thromboembolism; PE: pulmonary embolism; IQR: interquartile range; SD: standard deviation. P values were calculated using the Student’s t-test for normally distributed continuous variables, the Mann–Whitney U test for non-normally distributed variables, and the chi-square or Fisher’s exact test for categorical variables. A two-sided P value <0.05 was considered statistically significant.

Characteristics	Total Patients (N=412)	Patients Undergoing Repeat CTPA (n=61)	Patients Not Undergoing Repeat CTPA (n=351)	P Value
Age (years), mean±SD	56.2±15.8	59.8±14.9	55.5±15.9	0.048
Female sex, n (%)	218 (52.9%)	34 (55.7%)	184 (52.4%)	0.645
BMI (kh/m^2^), mean±SD	29.4±6.7	30.1±6.9	29.2±6.6	0.332
Smoking, n (%)	91 (22.1%)	19 (31.1%)	72 (20.5%)	0.048
Hypertension, n (%)	169 (41.0%)	31 (50.8%)	138 (39.3%)	0.092
Diabetes mellitus, n (%)	138 (33.5%)	24 (39.3%)	114 (32.5%)	0.288
Cancer, n (%)	54 (13.1%)	16 (26.2%)	38 (10.8%)	0.001
Prior VTE, n (%)	39 (9.5%)	12 (19.7%)	27 (7.7%)	0.002
Time between visits (days), median (IQR)	—	10 (6–18)	—	—
Initial Wells score, mean±SD	2.8±1.6	3.4±1.5	2.7±1.6	0.003
Initial D-dimer positive, n (%)	295 (71.6%)	53 (86.9%)	242 (68.9%)	0.005
Initial CTPA positive for PE, n (%)	59 (14.3%)	13 (21.3%)	46 (13.1%)	0.069
Treatment initiated on first visit, n (%)	68 (16.5%)	17 (27.9%)	51 (14.5%)	0.008

The mean age of the study population was 56.2 years (SD, 15.8); patients who underwent repeat CTPA were older than those who did not (59.8 vs. 55.5 years, P = 0.048). There was no significant difference in sex distribution between the groups. Patients in the repeat CTPA group were more likely to be smokers (31.1% (n=19) vs. 20.5% (n=72), P = 0.048), have active cancer (26.2% (n=16) vs. 10.8% (n=38), P = 0.001), and have a prior history of venous thromboembolism (VTE) (19.7% (n=12) vs. 7.7% (n=27), P = 0.002). The mean Wells score at the initial visit was significantly higher in the repeat group (3.4 vs. 2.7, P = 0.003), and initial D-dimer positivity was more common (86.9% (n=53) vs. 68.9% (n=242), P = 0.005). The median time between visits for those undergoing repeat imaging was 10 days (interquartile range (IQR), 6-18).

Initial CTPA revealed PE in 59 patients (14.3%), with a higher, though non-significant rate in the repeat CTPA group (21.3% (n=13) vs. 13.1% (n=46), P = 0.069). Anticoagulation was initiated during the initial visit in 17 (27.9%) patients who later had a repeat scan, compared to 51 (14.5%) of those who did not (P = 0.008).

Imaging findings

Among all patients, 342 (83.0%) underwent CTPA at the initial visit. During the repeat visit, all 61 (100%) patients underwent CTPA. The rate of PE detection on repeat imaging was significantly higher than on initial imaging (34.4% (n=21) vs. 14.3% (n=59), P < 0.001). Segmental or subsegmental PEs were the most common findings. RV strain was observed more frequently on repeat CTPA (14.8% (n=9) vs. 5.1% (n=21), P = 0.006), and pneumonia or other incidental findings were also more commonly reported (18.0% (n=11) vs. 9.2% (n=38), P = 0.038) (Table 2).

**Table 2:** Comparison of initial and repeat CTPA results (N=412) CTPA: computed tomography pulmonary angiography; PE: pulmonary embolism; RV: right ventricular

CTPA Findings	Initial Visit (N=412)	Repeat Visit (n=61)
CTPA performed, n (%)	342 (83.0%)	61 (100%)
Positive for PE, n (%)	59 (14.3%)	21 (34.4%)
Main pulmonary artery, n (%)	11 (2.7%)	4 (6.6%)
Segmental branches, n (%)	30 (7.3%)	10 (16.4%)
Subsegmental branches, n (%)	18 (4.4%)	7 (11.5%)
Negative CTPA, n (%)	277 (67.2%)	40 (65.6%)
Inconclusive CTPA, n (%)	6 (1.5%)	0 (0%)
RV strain on CTPA, n (%)	21 (5.1%)	9 (14.8%)
Additional findings (e.g., pneumonia), n (%)	38 (9.2%)	11 (18.0%)

D-dimer testing and diagnostic accuracy

D-dimer testing was performed in all 412 (100%) patients at both visits. The median D-dimer level was higher at the repeat visit than at the initial visit (970 ng/mL (IQR, 510-1580) vs. 820 ng/mL (IQR, 430-1430), P = 0.104). Positive D-dimer results were significantly more frequent during repeat visits (90.2% (n=55) vs. 71.6% (n=295), P = 0.001). The sensitivity and NPV of D-dimer remained high at both visits (>95%), although specificity was low (32.7% (n=135) at initial and 28.4% (n=17) at repeat visits). The PPV of D-dimer was notably higher during the repeat visit (38.2% (n=21) vs. 19.3% (n=57)), suggesting greater diagnostic yield in that context (Table 3).

**Table 3:** Diagnostic performance and clinical utility of D-dimer (N=412) IQR: interquartile range; PE: pulmonary embolism; NPV: negative predictive value; PPV: positive predictive value

Parameter	Initial Visit (N=412)	Repeat Visit (n=61)
D-dimer tested, n (%)	412 (100%)	61 (100%)
D-dimer level (ng/mL), median (IQR)	820 (430–1430)	970 (510–1580)
D-dimer positive, n (%)	295 (71.6%)	55 (90.2%)
Sensitivity for PE (%)	96.6	95.2
Specificity for PE (%)	32.7	28.4
NPV (%)	98.6	97.5
PPV (%)	19.3	38.2

Clinical outcomes and resource utilization

Patients undergoing repeat CTPA had significantly higher rates of hospital admission (34 of 61 patients (55.7%) vs. 59 of 351 patients (16.8%), P < 0.001) and ICU admission (6 of 61 patients (9.8%) vs. 5 of 351 patients (1.4%), P = 0.002). Management changes based on repeat CTPA findings occurred in 26 of 61 patients (42.6%), and anticoagulation was initiated in 21 of 61 patients (34.4%). Contrast-related adverse events, including contrast-induced nephropathy and mild allergic reactions, occurred more frequently in the repeat imaging group (4 of 61 patients (6.6%) vs. 4 of 351 patients (1.1%), P = 0.017). The 30-day all-cause mortality rate was low overall (7 of 412 (1.7%)), with no statistically significant difference between groups (Table 4).

**Table 4:** Clinical outcomes and utilization metrics (N=412) CTPA: computed tomography pulmonary angiography; ICU: intensive care unit; SAR: Saudi Riyal P values were calculated using the chi-square or Fisher’s exact test for categorical variables and the Mann–Whitney U test for skewed continuous data. A two-sided P value <0.05 was considered statistically significant.

Outcome	Total Patients (N=412)	Patients Undergoing Repeat CTPA (n=61)	Patients Not Undergoing Repeat CTPA (n=351)	P Value
Admission after repeat visit, n (%)	93 (22.6)	34 (55.7)	59 (16.8)	<0.001
ICU admission, n (%)	11 (2.7)	6 (9.8)	5 (1.4)	0.002
Adverse events (contrast-related), n (%)	8 (1.9)	4 (6.6)	4 (1.1)	0.017
30-day mortality, n (%)	7 (1.7)	2 (3.3)	5 (1.4)	0.289
Cost per patient (SAR), median (range)	3,850 (2,200–7,900)	6,950 (4,800–11,100)	3,000 (1,950–5,200)	<0.001

## Discussion

In this retrospective cohort study of ED patients undergoing evaluation for suspected PE, we found that repeat CTPA performed within 30 days of an initial negative or inconclusive workup yielded a significantly higher rate of PE diagnoses compared to the initial scans (34.4% vs. 14.3%, P < 0.001). These repeat evaluations were also associated with higher clinical severity, including increased ICU admissions and treatment escalation, but they came at the cost of greater resource utilization and a higher incidence of contrast-related adverse events. D-dimer testing, though highly sensitive at both time points, remained poorly specific, contributing to frequent imaging and diagnostic uncertainty.

Our findings underscore a critical tension in the diagnostic pathway for PE: the need to balance the risk of missed diagnoses against the harms of repeat imaging. The high rate of positive findings on repeat CTPA in the current study suggests that a subset of patients, particularly those with elevated pretest probability, active malignancy, prior VTE, or worsening symptoms, may indeed benefit from reimaging. The observed increase in PE severity and RV strain on repeat scans reinforces the possibility that some patients may have been initially missed or developed new thromboembolic events, raising the question of whether initial diagnostic pathways were sufficiently sensitive or complete [6-10].

At the same time, more than half of repeat scans were negative, and many were performed in the absence of major clinical deterioration. D-dimer testing was nearly universally positive at repeat presentation (90.2%), but specificity remained low (28.4%), leading to further diagnostic ambiguity. This supports prior literature showing that D-dimer has limited value in patients with recent illness, ongoing inflammation, or recurrent evaluation for thromboembolism [11-14]. In such cases, reliance on D-dimer may trigger unnecessary imaging rather than refine decision-making.

Importantly, patients undergoing repeat CTPA experienced significantly higher rates of hospital and ICU admission and incurred nearly double the median healthcare cost compared to those who did not undergo repeat imaging. Whether this reflects the underlying severity of illness or the consequences of further diagnostic escalation is unclear. Regardless, the findings highlight the need for improved risk stratification tools, potentially incorporating dynamic clinical scoring systems, age-adjusted or post-hoc D-dimer thresholds, and validated decision support algorithms, to identify patients for whom repeat imaging is most appropriate [12,15].

Limitations

Our study has several limitations. As a retrospective analysis, it is subject to residual confounding and limited by documentation quality. We could not assess all potential indications for repeat imaging, including changes in symptomatology or clinical judgment that may have influenced ordering decisions. Moreover, the single-region setting may limit generalizability, although our multicenter design and inclusion of real-world ED workflows strengthen external validity. Finally, while we tracked short-term outcomes, long-term sequelae such as chronic thromboembolic disease were not assessed.

## Conclusions

This study demonstrates that repeat pulmonary angiography in the emergency setting identifies a clinically significant proportion of missed or newly developed PEs, particularly among patients with elevated risk profiles. However, this increased diagnostic yield must be balanced against the substantial rise in healthcare utilization, adverse events, and costs associated with repeat imaging. Optimizing patient selection through enhanced risk stratification and judicious use of D-dimer testing may reduce unnecessary repeat scans, improve clinical outcomes, and promote more efficient resource use in the evaluation of suspected PE.
